# The clinical relevance of animal models in Sjögren’s syndrome: the interferon signature from mouse to man

**DOI:** 10.1186/s13075-015-0678-2

**Published:** 2015-07-03

**Authors:** Naomi I Maria, Petra Vogelsang, Marjan A Versnel

**Affiliations:** Department of Immunology, Erasmus Medical Center, Wytemaweg 80, 3015 CN Rotterdam, The Netherlands; Broegelmann Research Laboratory, Department of Clinical Science, University of Bergen, Jonas Lies vei 87, N-5021 Bergen, Norway

## Abstract

Mouse models have been widely used to elucidate the pathogenic mechanisms of human diseases. The advantages of using these models include the ability to study different stages of the disease with particular respect to specific target organs, to focus on the role of specific pathogenic factors and to investigate the effect of possible therapeutic interventions. Sjögren’s syndrome (SS) is a systemic autoimmune disease, characterised by lymphocytic infiltrates in the salivary and lacrimal glands. To date, effective therapy is not available and treatment has been mainly symptomatic. Ongoing studies in murine models are aimed at developing more effective and targeted therapies in SS. The heterogeneity of SS will most probably benefit from optimising therapies, tailored to specific subgroups of the disease. In this review, we provide our perspective on the importance of subdividing SS patients according to their interferon signature, and recommend choosing appropriate mouse models for interferon-positive and interferon-negative SS subtypes. Murine models better resembling human-disease phenotypes will be essential in this endeavour.

## Introduction to Sjögren’s syndrome

Sjögren’s syndrome (SS) is a systemic autoimmune disease characterised by lymphocytic infiltrates in salivary and lacrimal glands, sialadenitis and dacryoadenitis respectively. The disease can occur alone − primary Sjögren’s syndrome (pSS) − or together with other systemic autoimmune diseases such as systemic lupus erythematosus (SLE), systemic sclerosis or rheumatoid arthritis − secondary SS. The prevalence of pSS is estimated to be between 0.05 and 1%, with a ninefold predominance in females. Characteristic symptoms are dry eyes (keratoconjunctivitis sicca) and mouth (xerostomia), with frequent presence of multiple extraglandular manifestations, such as vasculitis, severe fatigue and multiorgan involvement [1–3]. At present, no common evidence-based intervention therapy is available and treatment is mainly symptomatic. Thus, further unravelling the pathophysiology of pSS is essential for finding novel biomarkers and identifying new treatment targets.

Murine models are a sophisticated way to model complex pathogenic mechanisms for diseases such as SS, despite discrepancies in the immune system between human and mouse [4]. These models provide the opportunity to manipulate disease processes and look at multiple organs in depth during the early disease state as well as disease progression, which is particularly difficult to achieve in humans but is essential in developing new therapeutic strategies. Recently, an extensive update on SS-like murine-models was published in this journal [5].

Here we focus on the present knowledge of the pathogenesis of human pSS with special regard to the interferon (IFN) signature. As IFN type I is a suggested key pathogenic factor, we discuss how the currently used mouse models fit with subdividing patients into IFN-positive and IFN-negative subgroups. Taking this subdivision as a starting point we also recapitulate relevant disease features and interventional studies in the nonobese diabetic (NOD) mouse model, which is the most commonly used SS mouse model.

## Sjögren’s syndrome: from man to mouse

Over the past decade, vast evidence for a role of IFN type I in the pathogenesis of pSS has emerged. We and others have described the presence of an IFN type I signature, assessed as upregulation of a distinct set of IFN type I inducible genes [6–12]. We describe the prevalence of the systemic IFN type I signature in over one-half of pSS patients (referred to as IFN-positive pSS), identifying a subgroup of patients with higher European League Against Rheumatism Sjögren’s Syndrome Disease Activity Index (ESSDAI) scores, presence of anti-Sjögren’s syndrome-associated autoantigen SSA (Ro52 and Ro60) and/or anti-Sjögren’s syndrome-associated autoantigen SSB (La) autoantibodies, higher immunoglobulin (Ig) G and B-cell activating factor (BAFF) levels, and lower C3 complement levels [10]. The abundant presence of autoantibodies leading to circulating immune complexes (ICs) is thought to be the main trigger inducing the IFN signature in pSS and SLE [13].

**Fig. 1:**
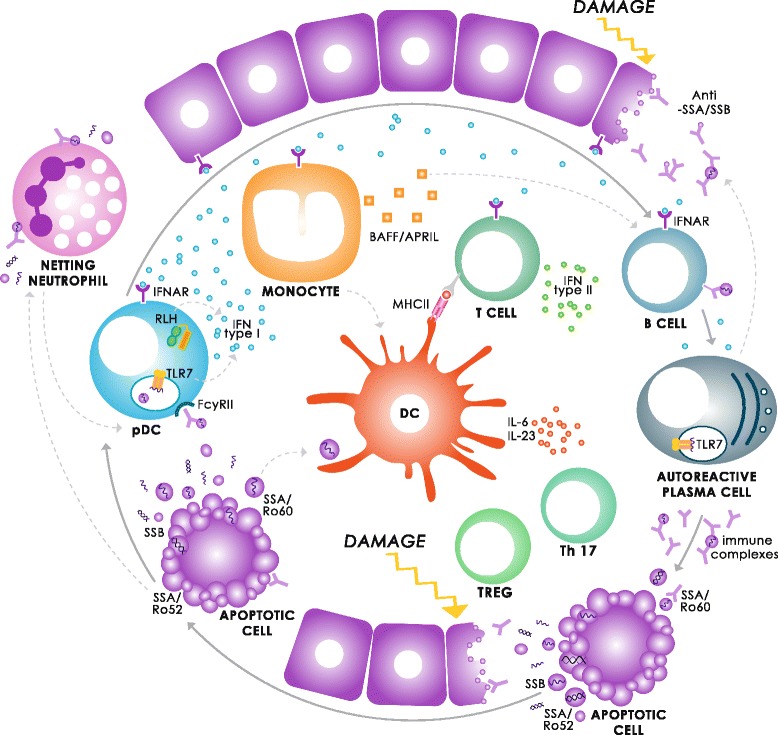
Multifactorial pathogenesis of primary Sjögren’s syndrome: interferons as culprits in the self-amplifying pathogenic loop. A damage trigger such as stress or infection leads to accumulating apoptotic debris, inducing rapid interferon (IFN) type I production by plasmacytoid dendritic cells (pDCs). IFN type I then binds to IFNα,β receptor (IFNAR) on adjacent target cells, which induces an IFN signature and IFN-primed mature effector cells, amongst others, by perpetuating the TLR7 pathway in autoreactive plasma cells as well as self-amplification in the pDCs. TLR7 upregulation in autoreactive plasma cells increases RNA-associated autoantibody production (SSA/Ro52, SSA/Ro60 and SSB/La). These RNA-associated autoantibodies form immune complexes together with self-apoptotic debris, further triggering the TLR7 pathway. Prolonged inflammation can lead to exhaustion of the complement system with decreased complement-mediated solubilisation and further accumulation of immune complexes. Neutrophils can cause further tissue damage by forming neutrophil extracellular traps, these netting neutrophils also being potent inducers of IFN type I production. Autoantibodies induce netting of IFN-primed neutrophils, further amplifying the loop. IFN-primed dendritic cells (DCs) activate T cells, as well as natural killer and natural killer T cells (data not shown), to produce vast amounts of IFN type II (IFNγ). Although TLRs are widely considered the usual suspects in autoimmune pathophysiology, recently the cytoplasmic RIG-I-like family of helicases (RLHs), RIG-I (DDX58) and MDA5 (IFIH1) have been gaining the spotlight as co-conspirators. Evidence points towards a collaborative effort between TLRs and RLHs, together enhancing inappropriate self-recognition and sustained IFN overactivation. IFIH1 upregulation has been identified in IFN-positive pDCs of primary Sjögren’s syndrome patients (unpublished data), as previously in glands of Sjögren’s syndrome-like (C57BL/6.NOD-Aec1Aec2) mice [61]. This IFN-driven pathogenic loop in primary Sjögren’s syndrome, in part driven by aberrant sensing of nucleic acids, can potentially lead to functional decline or even loss of function in target tissues. APRIL, a proliferation inducing ligand; BAFF, B-cell activating factor; IL, interleukin; MDA5, melanoma differentiation-associated protein 5; MHC, major histocompatibility complex; RIG-I, retinoic acid-inducible gene 1; Th17, T-helper type 17; TLR, Toll-like receptor; Treg, regulatory T cells

In addition to IFN type I, novel evidence indicates IFN activation in SS glands to be partly attributed to IFN type II activity (IFNγ) [14]. Whether this also holds true for systemic IFN activation in pSS remains to be established. A recent study in SLE, however, revealed distinct systemic type I and type II signatures [15]. Fig. 1 depicts the multifactorial pathogenesis of pSS, where IFNs are centred as the main culprits in the self-amplifying pathogenic loop. Better understanding of these distinct IFN pathways is crucial in selective therapeutic targeting.

Here we provide our perspective on the importance of subdividing pSS patients according to their IFN signature. To better resemble the human situation we will need to find models best fitting our subdivision, where one can be used as an IFN-negative model and the other, possibly accelerated by triggering the IFN system, can be used as its IFN-positive counterpart.

## Interferon-related murine models as interferon-positive Sjögren’s syndrome

As IFNs play an eminent role in human pSS pathogenesis, we studied the NOD mouse model for the presence of systemic IFN activation and found no indication for the presence of a systemic IFN signature (McGuiness B, Beumer W and colleagues, unpublished work). We therefore suggest the NOD mouse to be systemically IFN-negative, and set out to look at some previous studies in murine models for a possible IFN-positive counterpart. Many systemic autoimmune models used for SLE, amongst other diseases, extensively direct their attention to IFNs and their role in autoimmune development [13, 16–18]. Here we highlight existing autoimmune murine models which in our opinion are models to be revisited for potential SS-like disease.

### Interferon-inducible models in murine autoimmunity

External triggering of the IFN system in order to mimic systemic IFN overactivation is an approach often used in autoimmune-prone mice. Interestingly, the group of Deshmukh and colleagues applied this strategy to the lupus-prone New Zealand Black/White F1 mouse strain to study SS-like disease. After treatment of the mice with Toll-like receptor (TLR)-3 agonist poly(I:C), chronic systemic activation accelerated sialadenitis [19]. Interestingly, in addition to TLR3, poly(I:C) also engages the nucleic acid sensors retinoic acid-inducible gene I (RIG-I; DDX58) and melanoma differentiation associated antigen 5 (MDA5; IFIH1). These retinoic acid-inducible gene-like helicases have recently been suggested to collaborate with endosomal TLRs in amplifying IFN overactivation (see Fig. 1) [20–22], and thus might also contribute to overactivation of IFN in the poly(I:C)-treated mice. Retinoic acid-inducible gene-like helicases might be relevant therapeutic targets.

New Zealand Mixed (NZM) 2758 mice were additionally demonstrated to develop SS-like disease, after prior triggering of innate immunity by alum and induction of antibodies by immunisation with Ro52. This recent study focused on Ro52-induced salivary gland dysfunction and hypothesised that autoantibody deposition in the glands might be crucial to induce xerostomia and SS-like disease [23]. However, the role of IFNs in disease induction was not assessed.

### Imbalanced Toll-like receptor signalling leads to murine autoimmunity

For the generation of autoantibodies by B cells and IC-mediated IFN production by plasmacytoid dendritic cells, myeloid differentiation primary response gene 88 (MyD88)-dependent endosomal TLR7 and TLR9 are crucial. Nucleic acid-sensing TLRs such as TLR3, TLR7, TLR8 and TLR9 are located in intracellular endosomal compartments, consequently minimising possible exposure to self-antigens [24]. Intriguingly, our group recently found that the TLR7 pathway was upregulated in peripheral blood cells of IFN-positive pSS, whereas TLR9 was not (NI Maria and colleagues, unpublished work). A similar imbalance in TLRs was observed in murine lupus models. Opposing effects were described for TLR7 and TLR9: deletion of TLR7 limited autoimmunity, whereas TLR9 deletion paradoxically exacerbated disease. TLR7 deletion prevented RNA-associated antibody formation, whereas TLR9 deletion resulted in increased systemic inflammation and IC-induced glomerulonephritis [18]. That controlling TLR7 expression is essential in restricting autoimmunity already became clear when TLR7 gene duplication was demonstrated to be the sole requirement for accelerated autoimmunity in B6.Yaa mice. A substantial TLR7 increase even caused fatal acute inflammatory pathology and extensive dendritic cell dysregulation [16]. This mouse model, portraying the Yaa phenotype (TLR7 gene duplication; BXSB/MpJ-Yaa), was recently described to develop autoimmune dacryoadenitis in a study focusing on SS-like features [25], showing ample reason for revisiting the herein-mentioned autoimmune models for SS.

The imbalance in endosomal TLRs, which results in much more prominent lupus-like disease compared with wild-type (WT) mice, was also recently shown using mice deficient in TLR8 and/or TLR9. TLR8 deficient mice (B6.TLR8^−/−^) and particularly double TLR8/9-deficient mice (B6.TLR8/9^−/−^) displayed marked induction of TLR7, which was associated with more severe disease. This study concludes that both TLR8 and TLR9 act together in controlling TLR7 function, TLR8 particularly controlling TLR7 function in dendritic cells and TLR9 restraining TLR7 response in B cells [17].

Other potential TLR-related models to be revisited are reviewed elsewhere [13], elaborating on possibilities for exacerbated renal disease in TLR9^−/−^ versus reduced disease in their TLR7^−/−^ counterpart. TLR9-deficient autoreactive B cells no longer undergo class switching to pathogenic immunoglobulin isotypes (IgG2a and IgG2b), these TLR9^−/−^ mice having significantly smaller IgG deposits in the glomeruli and a prolonged survival compared with their TLR9 sufficient littermates. In contrast, TLR7-deficient mice no longer produce RNA-specific autoantibodies, developing less severe clinical disease than their TLR7 sufficient littermates [13].

Further investigating this imbalanced endosomal TLR signalling in IFN-driven pSS is warranted because most studies focus on SLE. The above mentioned murine models will be crucial in this endeavour, as the tight regulation of TLR7 is preventing autoreactivity leading to autoimmunity.

### Toll-like receptor inhibitors in interferon-related autoimmunity

The antimalarial agent hydroxychloroquine has long been considered an effective treatment for SLE and is frequently used for pSS. Chloroquines are said to block TLR7/9 activation either by preventing acidification and maturation of the endosomes or by interacting with nucleic acids and thereby preventing TLR triggering. The exact mechanisms and effects of chloroquines remain controversial (reviewed in [24]), and the effect of other TLR-blocking agents needs to be evaluated. In fact, a recent clinical trial evaluating the efficacy of hydroxychloroquine for the main symptoms of pSS concluded that 24 weeks of treatment with hydroxychloroquine did not improve symptoms compared with placebo. As the authors conclude, further studies are indeed warranted to determine longer-term outcomes of hydroxychloroquine use [26].

In a recent study, New Zealand Black/White F1 mice were treated with a TLR7/8/9 antagonist. Treated mice had lower serum autoantibody levels, reduction in proteinuria and kidney histopathology compared with their untreated counterparts [27]. Interestingly, small-molecule dual TLR7/9 antagonists are currently being tested in SLE patients; however, selective TLR7 inhibition might be a more promising approach and should, in our opinion, be evaluated in SS-like murine models for therapeutic efficacy.

### Targeting interferons and the IFNα,β receptor

The IFN type I family of cytokines comprises 17 subtypes, all signalling through a common receptor, the IFNα,β receptor (IFNAR). Blocking one single subtype with specific monoclonal antibodies has not shown the promising results anticipated in SLE trials, thus directly blocking the IFNAR might prove more beneficial. This approach is supported by a study showing that deletion of *Ifnar1* prevented severe disease in at least two lupus-prone strains. Interestingly, monoclonal antibodies against human IFNAR are currently tested for effectivity in SLE and systemic sclerosis [28, 29]. Recently, B6.IFNAR^−/−^ mice and WT mice triggered with IFN-inducing poly(I:C) were tested for salivary gland hypofunction. Loss of glandular function was evident in WT mice and limited in IFNAR^−/−^ mice, thus indicating a crucial role for IFN type I in xerostomia [30]. This observation points towards possible therapeutically beneficial effects in blocking the IFNAR in pSS.

Type I and type II IFNs signal via different receptors but share overlapping patterns of activated genes downstream. A recent study in human salivary gland tissue provided evidence that IFN type II (IFNγ) also contributes to the IFN signature in SS glands, and therefore the role of IFNγ needs to be further investigated in humans and mice. Interestingly, Ro60 peptide-immunised Balb/c mice significantly developed increased levels of IFNγ which correlated to decreased salivary flow [31], but the effect of increased IFNγ on a systemic IFN signature remains to be analysed.

### Autoantibodies and the BAFF/APRIL system in murine autoimmunity

IFN type I itself has vast effects on B-cell survival, possibly perpetuating the pathogenic loop. IFNs induce BAFF and a proliferation inducing ligand (APRIL) expression in monocytes, thereby contributing to antibody-producing plasma cell survival resulting in prolonged pathogenic autoantibody production. This further triggers IFN signalling as well as increased IC deposition in target tissues leading to chronic inflammation, damage and, ultimately, loss of function [32]. A recent study looking into the necessity of individual BAFF receptors BCMA, TACI and BR3 in receptor-deficient NZM 2328 mice concluded that any single BAFF receptor could be dispensable for lupus development in their model [33]. An earlier study by the same group however, found that BAFF-deficient NZM 2328 mice were largely spared from clinically overt disease. These mice only showed serological autoimmunity and renal pathology, whereas severe proteinuria and mortality were greatly reduced. Blocking BAFF might thus not fully reverse or eliminate underlying pathology, but seemingly leads to substantial clinical improvement in murine autoimmunity [34].

The study by Mackay and colleagues illustrates that our view on revisiting lupus-prone mouse models for SS pathology is a rewarding approach. In BAFF transgenic mice, considered a model for SLE, they observed SS-like disease characterised by severe sialadenitis, decreased saliva production and destruction of submandibular glands [35].

Supporting an important role for autoantibodies in pSS is a recent study performed in mice lacking the SLE and pSS autoantigen Ro52/TRIM21. After local damage induction by ear tagging, Ro52/TRIM21^−/−^ mice develop a lupus-like phenotype. Ro52/TRIM21 interacts with IFN regulatory factors that play a role in tightly regulating IFN signalling. In these mice, cellular damage drives pathology via potential triggering of endosomal TLRs through enhanced IFN production [36]*.* Ro52-targeted autoantibodies produced by autoreactive B cells might be interfering with the important regulatory role of Ro52/TRIM21 in maintaining balanced IFN signalling.

### Biologicals targeting the BAFF/APRIL system

Observations in lupus-prone mice led to rapid development of biologicals interfering with the BAFF/APRIL system such as belimumab (anti-BAFF) and atacicept (dual BAFF/APRIL inhibitor) [37]. Treatment of mice with rapidly progressive glomerulosclerosis in both early and late stages of disease with BAFF-Ig or TACI-Ig revealed that selective BAFF blockade was sufficient to both prevent disease development and progression. This led to the conclusion that both treatments were equally effective in retaining remission by prolonged B-cell depletion and a decrease in inflammatory response to renal IC deposition [38]. However, when disease was accelerated by IFNs, BAFF blockade only proved beneficial in the initiation phase and did not prevent progression once autoantibodies were present. These biologicals might not be sufficiently effective in later disease stages in autoantibody-positive patients and additional therapies targeting the pathways activated by IFN might be essential additives. Presently, belimumab is tested in human pSS, showing encouraging results. The belimumab in patients with pSS BELISS trials justify future studies with the BAFF-targeting drug in the autoantibody-positive subset of pSS [39, 40].

## The nonobese diabetic mouse model as interferon-negative Sjögren’s syndrome

### Nonobese diabetic and NOD-derived strains as spontaneous models for Sjögren’s syndrome

Apart from spontaneously developing diabetes, female NOD mice recapitulate typical SS-like symptoms such as decreased salivary flow and lymphocytic infiltrates in salivary glands [41, 42]. In contrast to inducible models for autoimmunity, NOD mice follow a pattern of initial morphological changes in the salivary glands prior to the onset of focal infiltration and manifestation of clinical symptoms at about 16 weeks of age [43, 44]. Furthermore, the disease profile in NOD mice resembles human SS concerning composition of infiltrates in salivary glands and partially in terms of the autoantibody profile [45]. Additionally, NOD mice develop lymphocytic infiltrates in the lacrimal glands and interestingly this dacryoadenitis develops more frequently in males. Microarray analysis on lacrimal glands of male NODs resulted in the identification of cathepsins as candidate biomarkers for SS [46, 47]. Initial infiltrating immune cells were found to be responsible for this increased cathepsin expression, thereby initiating lacrimal gland remodelling and degradation [48]. Interestingly, cathepsin expression increased in parallel with proinflammatory cytokines during autoimmune development in the male NODs [46]. In general, however, studies on lacrimal glands in NOD mice are rare and truly deserve more attention.

Autoimmune manifestations in NOD mice develop through a complex interplay of several factors composed of genetic predisposition and intrinsic immune dysfunctions which manifest under the influence of environmental conditions [49]. NOD mice develop diabetes in a major histocompatibility complex (MHC) class II-dependent manner prior to autoimmune exocrinopathy. Changing the MHC class II haplotype can protect NOD mice from developing diabetes but not SS-like disease, and can affect the severity of sialadenitis [50, 51]. Association of SS with genes encoding for human leukocyte antigen has been reported [52], and thus indicates the importance of the MHC haplotype.

Although T cells are the dominant type of lymphocytes found in the infiltrates, SS is thought to predominantly be a B-cell-mediated disease. NOD-Ig*μ*^null^ mice, which lack functional B cells, show the typical lymphocytic infiltrations in the salivary glands but do not develop hyposalivation until transfer of purified human SS-IgG or parental NOD-IgG, suggesting a crucial role for antibodies in an overt clinical stage of the disease rather than the initial phase of lymphocyte infiltration [53].

Transient depletion of regulatory T cells in NOD mice (performed at the age of 10 days) showed an accelerating effect on sialadenitis, while depletion in older mice did not influence sialadenitis [54]. Moreover, depletion of regulatory T cells in B-cell-deficient NOD^−/−^ mice reversed resistance to an autoimmune phenotype and increased both the presence and size of salivary gland infiltrations compared with WT NOD mice [55]. These findings underline the importance of a balanced regulatory-cell and effector-cell population in different stages of disease. Nevertheless, how depletion of regulatory T cells affects exocrine gland dysfunction remains to be established because the grade of infiltration does not correlate with actual dryness. This important point must be realised because there is also no correlation between gland function and grade of infiltration in humans. Studies combining analysis of gland function and infiltration pattern are therefore warranted.

The development of diabetes and SS-like disease in NOD mice is accepted to occur as two separate events, after identification of Idd3 (also termed autoimmune exocrinopathy 1, *Aec1*) and Idd5 (also termed autoimmune exocrinopathy 2, *Aec2*) as the genetic risk regions being sufficient for manifestation of exocrine dysfunction in NOD mice [56]. Introduction of Idd3 and Idd5 loci from NOD in the nonautoimmune prone C57BL/6 strain resulted in the so-called C57BL/6.NOD-Aec1Aec2 strain, which develops SS-like disease but not diabetes. Comparable with human SS, SS-like disease in C57BL/6.NOD-Aec1Aec2 mice is initiated by a preclinical silent phase before the onset of overt disease [57, 58]. A study comparing human SS parotid gland tissue with salivary glands of Aec2/Aec2 mice revealed common dysregulated pathways − associated with leukocyte recruitment and germinal centre formation [59] − underlining the relevance of the model for testing novel therapeutics.

### The interferon signature in NOD and NOD-derived strains

As described above, upregulation of IFN inducible genes termed the IFN signature has been observed both in target tissue and systemically in pSS patients [10, 60]. However, it is important to realise that observations in salivary glands do not imply that an IFN signature is systemically present.

The comparison of salivary gland IFN signatures in murine SS-like disease and human SS has been reviewed recently and clearly reveals expression of IFN type I induced genes in human pSS and NOD salivary glands [61, 62]. Interestingly, microarray analysis of male NOD lacrimal glands did not reveal increased IFN type I expression [46]. However, it is debatable whether data from murine glands can be compared with human peripheral blood studies. It is thus peculiar that most murine SS-like studies are focused on the IFN signature in the exocrine glands, fully disregarding the systemic aspect of the disease. We evaluated the gene expression patterns of NOD monocytes at various time points of disease development and found no evidence for systemic IFN type I activation (McGuiness B, Beumer W and colleagues, unpublished work). Hence, it should be mentioned that studies in human pSS simultaneously analysing the IFN signature in salivary glands and peripheral blood are still lacking. Therefore, there may well be pSS patients with local IFN type I activation in the glands but lacking systemic IFN type I activity, similar to NOD mice.

The pathogenic effect of IFN type I was assessed in C57BL/6.NOD-Aec1Aec2 mice deficient for *Ifnar1* (B6.*Aec1Aec2Ifnr1*^*−/−*^) and thus unable to respond to IFN type I-mediated signalling [63]. B6.*Aec1Aec2Ifnr1*^*−/−*^ mice did not upregulate IFN-responsive genes in the submandibular glands in comparison with their WT counterparts, although only a limited number of targets were tested in this study. Most strikingly, these mice were protected from salivary gland dysfunction and showed reduced infiltration in salivary and lacrimal glands, even though they still generated a robust systemic autoantibody response. Taken together, these findings support the hypothesis that systemic and local IFN activation can occur as two separate events.

Although type I IFNs were initially thought to be the driving force for the IFN signature, both IFN type I and type II activity were detected in human pSS salivary gland biopsies [14], while in pSS a contribution of type II to the systemic IFN signature remains to be established. In submandibular glands of NOD mice it has clearly been demonstrated that IFN type II/IFNγ participates in the early onset of SS-like disease. *NOD.IFNγ*^−/−^ and *NOD.IFN-γR*^−/−^ mice fail to develop sialadenitis, although retaining lymphocytic infiltrates in the lacrimal glands [64]. Nevertheless, to date these findings remain limited to local similarities. Thus, although there are many overlapping features between human pSS and NOD mice, evidence for a systemic IFN type I signature as observed in more than 50% of the human pSS patients is lacking in NOD mice. We conclude that NOD and NOD-derived strains rather represent the subset of systemically IFN-negative SS patients, and could contribute significantly to insights into the pathogenesis of IFN-negative pSS patients.

### Interventional studies in NOD and NOD-derived strains

Over the past years, several intervention studies have been performed on NOD or NOD-derived models, most of them using C57BL/6.NOD-Aec1Aec2 as a model for SS. Owing to space limitations, we selected from the large number of studies those that were tested in clinical trials or are in our view interesting candidates for translation into clinics.

Development of malignant lymphomas is a risk in pSS and therefore studies on mechanisms of malignant transformation are important. Lymphoid structure formation was found to be predictive for lymphoma development in human pSS [65]. To evaluate the inhibitory effect on lymphoid structure formation in exocrine glands of NOD mice, blockage of the lymphotoxin-beta receptor (LTBR) pathway by injection of LTBR-Ig was performed. LTBR blockage ablated lymphoid structures and reduced salivary gland tissue degeneration and lacrimal gland pathology, whereas salivary flow was partially restored [66, 67]. Nevertheless, injection of LTBR-Ig was performed in an early disease stage, and thus the effect on disease progression when administered in a late, overt disease phase remains to be tested.

The manipulation of co-stimulatory mechanisms by local gene therapy gave less conclusive results. Attempts to inhibit CD40 ligation in NOD mice did not alter SS-like disease at all [68], whereas CTLA-4-IgG expression in C57BL/6.NOD-Aec1Aec2 mice improved sialadenitis and salivary gland function [69]. At present, CTLA-4-IgG is being tested in clinical trials.

Several gene therapy-based studies targeting different SS-related cytokines were performed. In C57BL/6.NOD-Aec1Aec2 mice, targeting of interleukin (IL)-17 production by T-helper (Th) 17 cells locally in the salivary glands led to improved SS-like disease, independent of the disease state at administration [70]. Accordingly, systemically administered gene therapy using IL-27 to inhibit Th17 activity was performed in C57BL/6.NOD-Aec1Aec2 mice in early and advanced disease stages. The treatment did not alter lymphocytic infiltration but resulted in less severe clinical disease manifestation [71]. Overall, targeting Th17 networks might be a useful treatment option.

Recently the role of the cytokine IL-7, elevated in human-SS, has been analysed using C57BL/6.NOD-Aec1Aec2 mice. IL-7 was found to enhance the Th1 response and to promote the development of SS-like disease, whereas IL-7 blockade had a disease-ameliorating effect. Furthermore, IL-7 was found to influence Th1 responses via IFNγ activation [72]. Interestingly, IL-7 and TLR7 seem to act synergistically on T-cell and B-cell activation. Considering the importance of TLR7 activation in autoimmunity, IL-7 should be considered a future candidate for intervention studies [73]. Besides TLR7, TLR9 seems to be involved in the pathogenesis of SS-like disease in NOD mice. Activation of TLR9-dependent p38 mitogen-activated protein kinase was recently found to occur in an early disease state [74]. However, agonistic TLR9 treatment that activated the alternative nuclear factor-κB pathway increased salivary flow [75]. These findings support a crucial role for the TLR7/9 balance, where increased TLR9 signalling potentially salvages autoimmunity whilst a tilt towards TLR7 signalling actually exacerbates autoimmunity.

Taking these results together, it is important to note that the stage of SS-like disease in NOD mouse models strongly influences the outcome of different therapeutic interventions. The majority of treatments tested in mice were performed at an early disease stage. Intervening when overt disease is present in relevant animal models is required before these approaches are applicable in humans. Besides, human SS is mostly diagnosed in an overt phase.

## Perspectives: from mouse to man

**Fig. 2:**
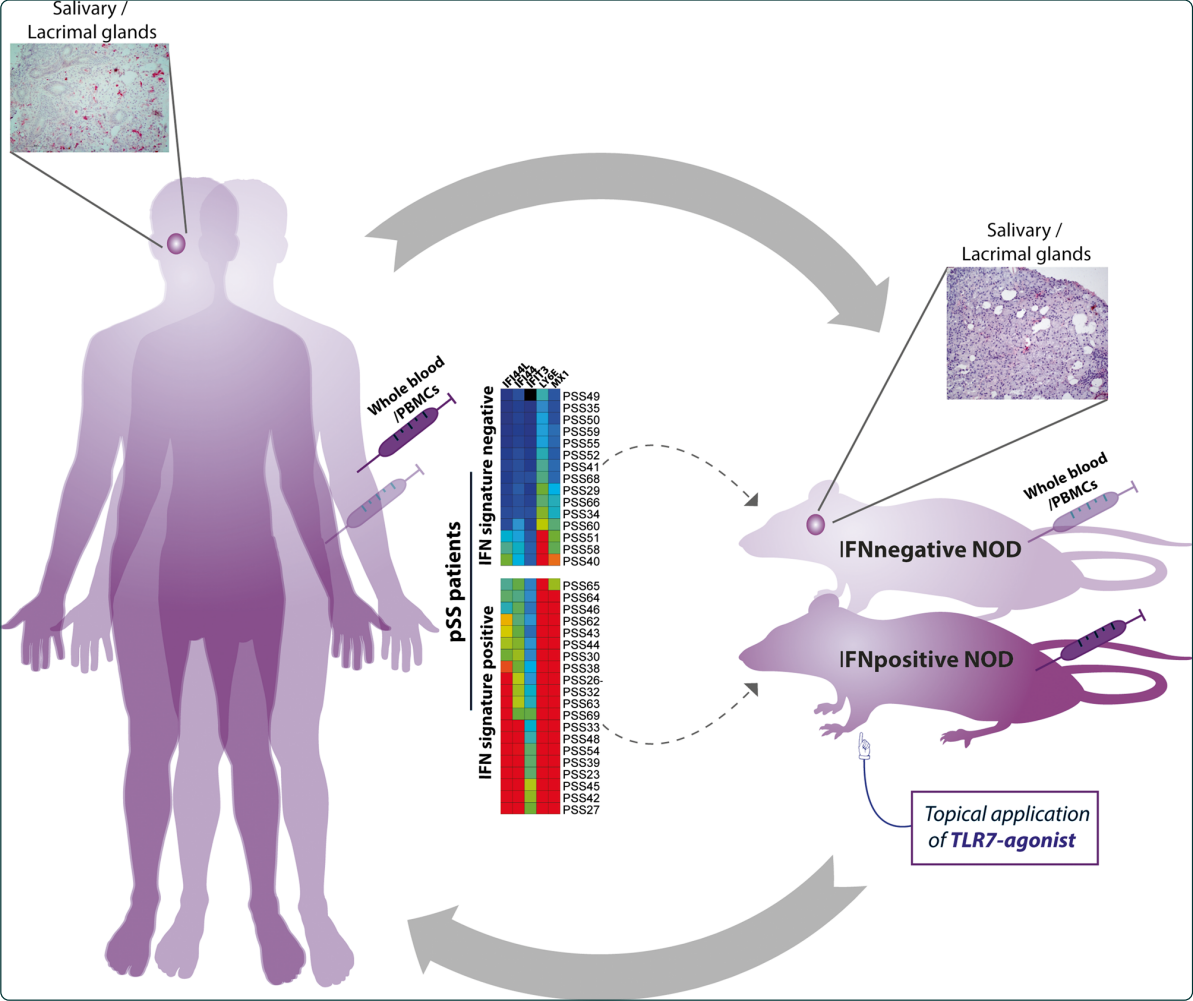
Primary Sjögren’s syndrome: a back and forth interplay from mouse to man. To investigate the heterogeneous and complex pathogenesis of primary Sjögren’s syndrome (pSS), murine models such as nonobese diabetic (NOD) or NOD-derived mice are indispensable. Knowledge-based implementation derived from mouse models is commonly implemented into human studies and potential clinical trials. As the heterogeneity of patients is often underestimated, we propose a back and forth interplay of knowledge between mouse and man, looping the circle from man to mouse and back. Hereby, models will further be improved to better resemble specific aspects of human disease, essential for both therapeutic development and outcome prediction. An important step will be to compare common deregulated pathways in both mouse and man to address therapeutic manipulations, by isolating whole blood/peripheral blood mononuclear cells (PBMCs) or extracting target tissue biopsies from salivary and lacrimal glands. Patient selection and subgrouping according to their interferon (IFN) signatures, into IFN-positive and IFN-negative subgroups, will require separate mouse models per subgroup. As the NOD mouse most probably represents the subset of systemically IFN-negative patients, we here propose the NOD model revisited: TLR7-induced systemic IFN signature in the NOD mouse as the IFN-positive counterpart, by topical application of the TLR-agonist imiquimod. Furthermore, comparing equal compartments in both subgroups of mice and men will give new insights into both the similarities and differences. Mouse models will remain crucial for preclinical exploration studies and will need continued revisiting and refining. TLR, Toll-like receptor

When drawing conclusions from mouse models for clinical relevance in humans, a proper understanding of both the similarities and differences in immune function between mouse and man remains crucial for adequate interpretation of mouse studies [4]. There is ongoing controversy when using murine models regarding appropriate extrapolation of obtained knowledge into a clinical setting. We therefore propose the best strategy to be a back and forth interplay between mice and men. Revisiting existing mouse models in a more disease-related way certainly might help to elucidate underrated aspects of the disease. In particular, lupus-prone models that can be used for SS might reveal novel insights into SS pathogenesis. Here we aimed to highlight this approach, looping the circle from mouse to man and back (see Fig. 2).

As SS in humans is very heterogeneous, multiple animal models are necessary to fully elucidate disease pathogeneses. Subtyping disease according to the prominent pathogenic player IFN, according to the IFN signature, is therefore a crucial first step. We highly recommend choosing appropriate mouse models for both IFN-negative and IFN-positive disease subtypes in future studies. In particular, we suggest NOD as a model for systemically IFN-negative SS. Recently, evidence for distinct roles of IFN type II and type III in autoimmunity has emerged [14, 15, 76], probably making subtyping even more complex in the near future but also much more effective for strategic therapeutic targeting.

In terms of developing new approaches for therapeutic interventions, the TLR–IFN network is a promising target and warrants more in-depth investigation. The delicate balance between endosomal TLRs appears crucial to prevent autoimmunity, and a better understanding of these pathways and how they are balanced will provide insight into specific targeted therapies [77]. Interestingly, tackling TLR7 will have effects on both plasmacytoid dendritic cells and B cells simultaneously, and might prove beneficial by inhibiting multiple aspects of the disease with one compound. Whether TLR7 blockade alone or in combination therapies will prevent and/or ameliorate pSS pathogenesis remains to be investigated [13].

## Conclusions

The heterogeneity of SS will most probably benefit from optimising therapies, tailored to specific subgroups of the disease. Here we provide our perspective on the importance of subdividing SS patients according to their IFN signature, and recommend choosing appropriate mouse models for IFN-positive and IFN-negative SS subtypes. Murine models better resembling human-disease phenotypes will be essential in this endeavour.

Several key messages can be obtained from this review:

1. Simultaneous histopathology and functional analysis of salivary and lacrimal glands in relevant mouse models will result in further insight into SS pathogenesis
2. Lupus-prone mouse models should be revisited for salivary and lacrimal gland pathology and dysfunction
3. A distinction between local and systemic presence of IFN activity is essential, because local IFN activation does not directly imply the presence of systemic IFN activation
4. Therapeutic interventions should be tested when overt disease is present in relevant animal models, because pSS is diagnosed in this phase in humans

## Abbreviations

Aec: autoimmune exocrinopathy
APRIL: a proliferation inducing ligand
BAFF: B-cell activating factor
ESSDAI: European League Against Rheumatism Sjögren’s Syndrome Disease Activity Index
IC: immune complex
IFN: interferon
IFNAR: IFNα,β receptor
Ig: immunoglobulin
IL: interleukin
LTBR: lymphotoxin-beta receptor
MHC: major histocompatibility complex
MyD88: myeloid differentiation primary response gene 88
NOD: nonobese diabetic mice
pSS: primary Sjögren’s syndrome
SLE: systemic lupus erythematosus
SS: Sjögren’s syndrome
Th: T-helper
TLR: Toll-like receptor
WT: wild type
